# Guideline-concordance along the cancer care continuum and breast cancer mortality by race and ethnicity: a SEER-Medicare study

**DOI:** 10.1007/s10552-025-02099-9

**Published:** 2026-01-21

**Authors:** Emma L. Herbach, Ryan M. Carnahan, Lauren E. McCullough, Bradley D. McDowell, Michaela Curran, Kai Wang, Ingrid M. Lizarraga, Mary E. Charlton, Sarah H. Nash

**Affiliations:** 1 Department of Epidemiology, College of Public Health, University of Iowa, Iowa City, IA USA; 2 Sylvester Comprehensive Cancer Center, University of Miami Miller School of Medicine, Miami, FL USA; 3 Department of Epidemiology, Rollins School of Public Health, Emory University, Atlanta, GA USA; 4 University of Iowa Holden Comprehensive Cancer Center, Iowa City, IA USA; 5 Department of Community and Behavioral Health, College of Public Health, University of Iowa, Iowa City, IA USA; 6 Department of Biostatistics, College of Public Health, University of Iowa, Iowa City, IA USA; 7 Department of Surgery, University of Iowa Carver College of Medicine, Iowa City, IA USA; 8 Iowa Cancer Registry, Iowa City, IA USA; 9 New Mexico Tumor Registry, Albuquerque, NM USA; 10 Department of Epidemiology, Biostatistics, & Preventive Medicine, University of New Mexico Comprehensive Cancer Center, Albuquerque, NM USA

**Keywords:** Breast cancer, Survival, Racial disparities, Guideline-concordant care

## Abstract

**Purpose:**

To examine the relationship between guideline-concordant breast cancer care and hazard of cancer death by patient race and ethnicity.

**Methods:**

We used SEER-Medicare data to identify 212,555 older women diagnosed with invasive breast cancer between 2000 and 2017. Guideline-concordant diagnostic workup, locoregional treatment, and initiation of systemic therapy were defined using NCCN guidelines. Hazards of breast cancer death 2 and 5 years from diagnosis by each guideline-concordance outcome overall and stratified by race and ethnicity were estimated using Cox proportional hazards models.

**Results:**

Non-concordant diagnostic workup, locoregional treatment, and systemic therapy initiation were each associated with increased hazards of 2- and 5-year breast cancer mortality (diagnostics HR_2-year_ (95% CI) 1.33 (1.25–1.41), HR_5-year_ 1.29 (1.23–1.35); locoregional HR_2-year_ 2.10 (1.98–2.23), HR_5-year_ 1.83 (1.76–1.90); systemics HR_2-year_ 1.67 (1.51–1.84), HR_5-year_ 1.56 (1.45–1.68)). Non-concordant diagnostic workup and systemic therapy initiation were associated with greater hazard of 2- and 5-year breast cancer death among Black, Asian/Pacific Islander, Hispanic White, and non-Hispanic White patients; there was no consistent association among American Indian/Alaska Native patients for either outcome. Locoregional treatment was strongly associated with hazards of cancer death for all groups.

**Conclusion:**

Equitable delivery of guideline-recommended breast cancer care from diagnosis through treatment across racial and ethnic groups may mitigate survival disparities. Efforts to improve access to high-quality care must be informed by and responsive to the social and structural root causes of health inequities.

**Supplementary Information:**

The online version contains supplementary material available at 10.1007/s10552-025-02099-9.

## Introduction

Black and American Indian and Alaska Native (AIAN) women experience disproportionately high breast cancer mortality [1, 2]. Conversely, Asian and Pacific Islanders (API) have the lowest incidence and mortality rates of any race and ethnicity group [1, 2]. These racial and ethnic differences in breast cancer mortality persist independently of stage, subtype or histology, age at diagnosis, and other prognostic factors [3–8]. Social and structural factors, including access to high-quality treatment, are emerging as fundamental causes of racial and ethnic survival disparities [9–19].

Clinical practice guidelines, such as those put forth by the National Comprehensive Cancer Network (NCCN), provide up-to-date recommendations based on clinical trial data and expert consensus [20]. Provision of care according to guidelines is associated significant reductions in mortality [21–25]. Black, AIAN, and Hispanic women are less likely to receive guideline-recommended breast cancer care compared to White women along the continuum from diagnosis through treatment and supportive care [13, 14, 17, 26–42]. Our previous work documented consistent disparities in receipt of guideline-recommended diagnostic workup, locoregional treatment, and systemic therapy initiation among Black and AIAN women compared to NHW, even while accounting for contextual factors related to where patients lived and sought care [43]. Facility characteristics (accreditation and volume) were associated with greater odds of guideline-recommended diagnostics and systemic therapy initiation. Black racial residential segregation was associated with lower odds of guideline-recommended locoregional and systemic treatments, while rurality and low socioeconomic status were associated with lower odds of guideline-recommended diagnostics. While some studies have examined the effects of treatment receipt and/or quality on survival disparities, few have concurrently considered diagnostic procedures necessary to determine the appropriate treatment. The goal of this investigation was to characterize the survival impact of high-quality care during the first phases of breast cancer care (i.e., diagnosis through first-line treatment initiation) across racially and ethnically diverse populations. Rather than using race and ethnicity as the primary predictor or confounding variable to adjust for, we opted for stratification in line with recommendations for antiracist health services research and measurement of structural racism [44–46]. The resulting research question investigates whether the survival benefits of high-quality care are equally impactful across racial and ethnic groups.

The primary aims of this study were to (1) evaluate the associations between guideline-concordant breast cancer care and hazards of cancer death and (2) examine whether this relationship varied between strata of race and ethnicity. Because the social and structural forces underlying racial and ethnic disparities in care are continuous and shape the entire cancer care continuum, we examined guideline-concordance at multiple time points—diagnosis, locoregional treatment, and initiation of systemic therapy—in relation to hazard of death at 2- and 5-year post-diagnosis.

## Methods

### Data source and study population

This retrospective cohort study used the linked Surveillance, Epidemiology, and End Results (SEER)-Medicare database containing tumor registry and claims data for patients diagnosed with breast cancer between 2000 and 2017 (RRID:SCR_025811). SEER registry data included demographic, tumor, and clinical information derived from medical records by trained abstracters [47, 48]. Vital status and cause of death (where applicable) reported by SEER are sourced from the National Center for Health Statistics data [47, 49]. The last date of follow-up for all data reported in the SEER registry files was 31 December, 2018. Medicare enrollment and fee-for-service claims data from 1999 to 2019 were analyzed; fee-for-service claims included inpatient and skilled nursing facility (MedPAR), outpatient (OUTPAT), and professional services National Claims History (NCH) files. Medicare Part D prescription drug event claims for patients with the corresponding coverage were also examined, with data available starting in 2007 [47].

**Fig. 1:**
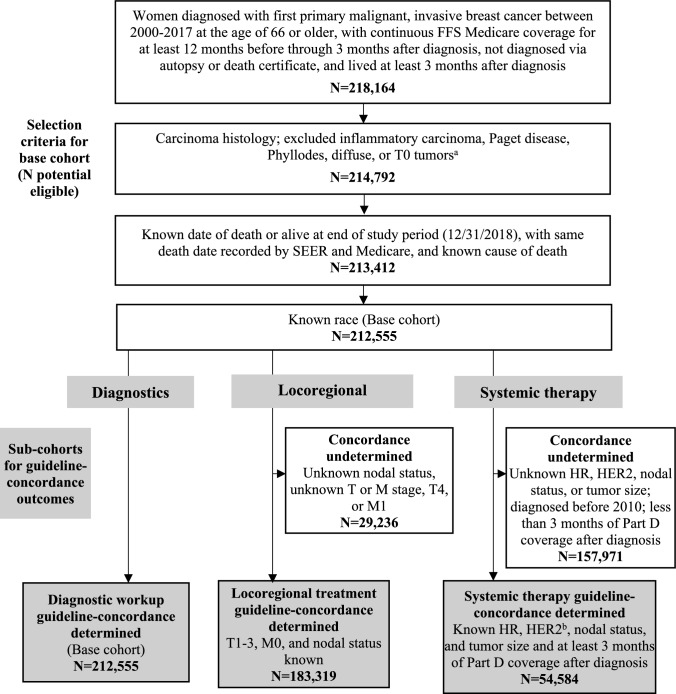
Flow diagram of cohort selection criteria. Figure created in Microsoft Word. *FFS* Fee-for-service. ^a^Histology and tumor types were identified using International Classification of Diseases for Oncology, 3rd edition (ICD-O-3) codes. The following codes were excluded: 8530, 8540-8543, 9020, and any code greater than 8589. ^b^HER2 status variable was first reported by SEER for 2010diagnoses. Patients not diagnosed in 2010 or later (when HER2 status variable is available) are excluded

Women diagnosed with a first invasive primary breast tumor between 2000 and 2017 at age 66 or older in a SEER-18 catchment area were eligible for inclusion (Fig. 1). Patients with T0, diffuse, non-carcinoma histologies, inflammatory carcinoma, Paget disease, or Phyllodes tumors were excluded. Eligible patients had continuous fee-for-service Part A and B coverage for at least 12 months prior to diagnosis through at least three months after diagnosis. Patients who died within three months of diagnosis and those with other or unknown race were excluded. Only patients with a known date and cause of death or who were alive at last follow-up (end of 2018) were eligible for inclusion. For patients who died during the study period, the date of death reported by SEER must match the date reported Medicare; those with discrepant death dates were excluded.

Patients meeting the above criteria constitute the “base cohort”—all of whom should have received diagnostic workup according to practice guidelines. Because locoregional treatment and systemic therapy regimens are determined by specific clinical characteristics, these outcomes were assessed among subsets of the base cohort who met additional criteria specific to each outcome (Table 1). Locoregional treatment was assessed among patients who were non-metastatic (M0) stage T1–T3 with known nodal status (based on TNM staging). Systemic therapy was assessed among patients with known tumor subtype, nodal status, and tumor size with Part D coverage diagnosed in 2010 or later. Part D prescription drug coverage was required for at least 3 months after diagnosis (same duration as fee-for-service) because most hormone therapies (and some other systemics) are obtained via pharmacies. The systemics cohort was further restricted to diagnoses 2010 or later, when complete tumor subtype data were available in SEER.

**Table 1:** Guideline-concordance outcome definitions

Guideline-concordance outcome	Cohort	Procedures included	Indication based on
Diagnostic workup	- Base cohort (all eligible patients)	- Diagnostic mammography - Pathology review - Determination of HR and HER2 status (only 2010 + diagnoses)	- Clinical M stage (M0, M1, or M unknown)
Locoregional treatment	- Stage T1-T3, M0, with known nodal status	- Axillary staging - Surgery - Radiation	- TNM staging - Tumor size - Age - Surgery type (for radiation only)
Systemic therapy initiation	- Known HR and HER2 status, nodal status, and tumor size	- Chemotherapy - Hormone therapy - Anti-HER2 therapy	- HR and HER2 status - Stage (summary stage I-IV or unknown) - Nodal involvement (N) - Tumor size

Procedures included in the definition of each guideline-concordance outcome and clinical characteristics used to determine indication for each component of care. Full description of the operational definitions for each guideline-concordance outcome are provided in Supplementary File 1 of our previous manuscript [46]

*HER2* human epithelial growth factor receptor 2, *HR* hormone receptor, *M* metastatic staging, *N* nodal involvement, *T* tumor staging, *TNM* Tumor, Nodes, and Metastasis

Men were not included in this study due to potential differences in biologic/clinical characteristics or patterns of care, partially owing to the rarity of the disease compared to women. Of note, NCCN guidelines for invasive breast cancer include special considerations for breast cancer in men, which acknowledge that treatment recommendations across genders are extrapolated from trials of women with breast cancer due to the underrepresentation of men in these trials [20]

### Study variables

Race and ethnicity data were obtained from Medicare enrollment files and the SEER Cancer File. Survival outcomes of interest were hazards of death from breast cancer at 2 and 5 years after diagnosis. Date and cause of death were obtained from the SEER data, derived from the National Center for Health Statistics data.

Guideline-concordant diagnostic workup, locoregional treatment, and systemic therapy were operationalized by adapting NCCN guidelines and established quality metrics, with concordance assessed according to tumor characteristics [20, 43, 50, 51]. Full operational definitions for guideline-concordance outcomes and identifying procedures with SEER and/or Medicare claims data are described in detail in our previous work [46, 51]. Briefly, guideline-concordance was defined as receipt of care as indicated or non-receipt of care that was not indicated across all procedures within the specified phase of care (Table 1**)**. Diagnostic procedures included diagnostic mammography, pathology review, and hormone receptor (HR) and human epithelial growth factor receptor 2 (HER2) status determination. HER2 status was only available for patients diagnosed in 2010 or later, so HER2 determination was not included in the definition of diagnostic concordance for patients diagnosed before 2010. Locoregional treatment included axillary staging, surgery, and radiation. Systemic therapy initiation—including chemotherapy, hormone therapy, and anti-HER2 therapy—was identified by the presence of at least one claim associated with the treatment and/or documentation of receipt in SEER (chemotherapy only).

Patients were classified as indicated, discretionary, or not indicated for each procedure based on tumor stage, subtype, and other clinical characteristics as defined in NCCN guidelines. Each guideline-concordance outcome was operationalized by examining indication for and receipt of all procedures within the specified phase of care. If a procedure was classified as discretionary, care was considered guideline-concordant regardless of whether it was performed. Outcomes were assessed among the sub-cohorts of patients eligible to receive that form of care, with sufficient data to characterize indication and receipt (Fig. 1). For example, only patients with known HR and HER2 status were included in systemic therapy analyses because this treatment is indicated by tumor subtype.

Covariates included year of diagnosis, age at diagnosis, subsequent malignant tumors diagnosed within 12 months of primary cancer diagnosis, marital status, low-income subsidy, and health status. The low-income subsidy covariate serves as a proxy for individual-level poverty, reflecting an individual’s income level and requisite out-of-pocket costs during the baseline (pre-diagnosis) period based on monthly enrollment data. Low-income subsidy was chosen over other variables such as Medicaid eligibility/enrollment due to variation in Medicaid eligibility across states. Health status variables included comorbidity and frailty, which were estimated using validated algorithms of fee-for-service claims during the 12 months before diagnosis. Comorbidity was operationalized using the National Cancer Institute (NCI) Comorbidity Index—an adaptation of the Charlson Comorbidity Index developed specifically for use with claims data from the SEER-Medicare linkage (RRID:SCR_025810) [52–56]. The NCI Comorbidity Index is a weighted continuous score reflecting the number and severity of comorbid health conditions (not including cancer) that may alter mortality risk, such as cardiovascular, pulmonary, neurologic, renal, liver, and rheumatologic diseases, diabetes, and AIDS. Frailty was operationalized using the claims-based index developed and tested on Medicare data by Kim et al. [57, 58]. The Kim Frailty Index is a continuous score that quantifies functional status and vulnerability to adverse health outcomes such as disability, hospitalization, and death among older adults [59].

### Statistical methods

We estimated hazard ratios (HRs) with 95% confidence intervals (CIs) via Cox proportional hazards models for breast cancer death 2 and 5 years from diagnosis by guideline-concordant diagnostics, locoregional treatment, and systemic therapy initiation, respectively. Follow-up time for each patient was calculated from the first day of the diagnosis month through death, end of study data (31 December, 2018), or end of continuous fee-for-service insurance coverage, whichever came first. To estimate survival at 2- and 5-year post-diagnosis, follow-up was capped at the corresponding endpoint. Deaths due to any cause other than breast cancer were censored.

Kaplan–Meier curves by each guideline-concordance measure were visualized as failure plots (1-survival probability) reflecting the cumulative probability of breast cancer death. The Cox proportional hazards assumption was assessed by examining Schoenfeld residuals and visually examining Kaplan Meier Failure Plots for proportionality across all outcomes (Online Resource 1). We observed no evidence of violation of the proportional hazards assumption. We opted to use the cause-specific hazard model over the Fine-Gray approach because Fine-Gray is less preferable for causal interpretations [60, 61].

We fit a series of multivariable-adjusted models. Model 1 was adjusted for year of diagnosis, stage at diagnosis, and age at diagnosis. Model 2 was additionally adjusted for tumor subtype. Model 3 covariates included health status (comorbidity and frailty scores), demographics (marital status and low-income subsidy), subsequent malignant tumors diagnosed within the treatment period, and Model 2 covariates. The final adjusted model, Model 4, adjusted for all guideline-concordance outcomes (other than the predictor of the model) in addition to covariates from the prior models. Modification of the relationship between guideline-concordance and cancer death by race and ethnicity was examined using stratification (i.e., separate regression models run for each racial and ethnic group). Secondarily, we assessed the statistical significance of the interaction between race and ethnicity and guideline-concordance with multiplicative interaction terms, which were otherwise identical to the multivariable-adjusted models in the primary analysis.

Two-tailed tests with a significance level of 0.05 were utilized for all analyses. Analyses were performed in SAS software version 9.4 (SAS Institute Inc., Cary, NC; RRID:SCR_008567). Data visualization was performed using R Statistical Software (v7.2.576; RRID:SCR_001905) via the ggplot2 R package (v2.0.6; RRID:SCR_014601) [62–64]. This project was approved by the University of Iowa Institutional Review Board.

## Results

In total, 212,555 women were eligible for this study. Non-Hispanic White women were the largest racial and ethnic group, comprising 83.3% (*n* = 177,012) of the study cohort. The next largest demographic was Black women at 7.6% (*n* = 16,187), followed by 4.6% Hispanic White (*n* = 9,863), 4.1% API (*n* = 8,616), and 0.4% AIAN (*n* = 877). The average age at breast cancer diagnosis was 76.1 years (standard deviation 7.1); AIAN women had the youngest average age at diagnosis of 74.7 years while non-Hispanic White women had the eldest average age of 76.3 years. Median follow-up time was 5 years (Interquartile Range 2.5–8.8 years). Among women who died from cancer, the median time to cancer death was 2.6 years (Interquartile Range 1.2–5.0 years). Cohort descriptives by race and ethnicity are provided in Table 2. Alternatively, descriptives by guideline-concordance are provided in Online Resource 2.

**Table 2:** Characteristics of study population by race and ethnicity

Covariate	Level	Overall *n* = 212,555	Black *n* = 16,187	American Indian/Alaska Native *n* = 877	Asian/Pacific Islander *n* = 8,616	Hispanic White *n* = 9,863	Non-Hispanic White *n* = 177,012
Death endpoints							
Deaths within 2 years after diagnosis	Alive	187,957 (88.4)	13,518 (83.5)	764 (87.1)	7,959 (92.4)	8,859 (89.8)	156,857 (88.6)
	Cancer death	12,348 (5.8)	1,538 (9.5)	63 (7.2)	331 (3.8)	544 (5.5)	9,872 (5.6)
	Other (non-cancer) death	12,250 (5.8)	1,131 (7.0)	50 (5.7)	326 (3.8)	460 (4.7)	10,283 (5.8)
Deaths within 5 years after diagnosis (or end of study data (12/31/2018), whichever comes first)	Alive	159,472 (75.0)	11,073 (68.4)	645 (73.6)	7,208 (83.7)	7,698 (78.1)	132,848 (75.1)
	Cancer death	22,775 (10.7)	2,610 (16.1)	111 (12.7)	641 (7.4)	1,029 (10.4)	18,384 (10.4)
	Other (non-cancer) death	30,308 (14.3)	2,504 (15.5)	121 (13.8)	767 (8.9)	1,136 (11.5)	25,780 (14.6)
Time to cancer death (years)	Mean (SD)	3.6 (3.2)	2.9 (2.7)	3.1 (2.8)	3.6 (3.0)	3.5 (3.0)	3.7 (3.2)
Follow-up time (years)	Mean (SD)	6.1 (4.4)	4.9 (4.0)	5.6 (4.2)	6.0 (4.3)	5.6 (4.2)	6.2 (4.4)
Tumor characteristics							
Stage at diagnosis	I	108,206 (50.9)	6,515 (40.3)	410 (46.8)	4,536 (52.7)	4,543 (46.1)	92,202 (52.1)
	II	62,206 (29.3)	5,450 (33.7)	267 (30.4)	2,619 (30.4)	3,155 (32.0)	50,715 (28.7)
	III	18,942 (8.9)	1,980 (12.2)	80 (9.1)	709 (8.2)	1,019 (10.3)	15,154 (8.6)
	IV	9,027 (4.2)	1,010 (6.2)	44 (5.0)	296 (3.4)	448 (4.5)	7,229 (4.1)
	Unknown/missing	14,174 (6.7)	1,232 (7.6)	76 (8.7)	456 (5.3)	698 (7.1)	11,712 (6.6)
Hormone receptor (HR) status	Positive	166,597 (78.4)	11,122 (68.7)	675 (77.0)	6,900 (80.1)	7,569 (76.7)	140,331 (79.3)
	Negative	25,609 (12.0)	3,210 (19.8)	114 (13.0)	1,112 (12.9)	1,289 (13.1)	19,884 (11.2)
	Borderline or unknown	20,349 (9.6)	1,855 (11.5)	88 (10.0)	604 (7.0)	1,005 (10.2)	16,797 (9.5)
Human Epidermal Growth Factor Receptor 2 (HER2) status	Positive	9,535 (4.5)	832 (5.1)	53 (6.0)	549 (6.4)	543 (5.5)	7,558 (4.4)
	Negative	76,455 (36.0)	5,810 (35.9)	376 (42.9)	3,765 (43.7)	3,914 (39.7)	62,590 (35.4)
	Borderline or unknown	7,328 (3.4)	637 (3.9)	47 (5.4)	333 (3.9)	418 (4.2)	5,893 (3.3)
	Data not available	119,237 (56.1)	8,908 (55.0)	401 (45.7)	3,969 (46.1)	4,988 (50.6)	100,971 (57.0)
Triple-negative subtype	Triple-negative	7,701 (3.6)	1,123 (6.9)	41 (4.7)	367 (4.3)	425 (4.3)	5,745 (3.3)
Age at diagnosis	Mean (SD)	76.1 (7.1)	75.6 (7.2)	74.7 (6.7)	75 (6.7)	74.9 (6.8)	76.3 (7.2)
Year of diagnosis	2000–2001	24,284 (11.4)	1,670 (10.3)	63 (7.2)	589 (6.8)	849 (8.6)	21,113 (11.9)
	2002–2003	24,056 (11.3)	1,788 (11.1)	84 (9.6)	726 (8.4)	891 (9.0)	20,567 (11.6)
	2004–2005	24,075 (11.3)	1,853 (11.5)	84 (9.6)	808 (9.4)	1,070 (10.9)	20,260 (11.5)
	2006–2007	23,553 (11.1)	1,768 (10.9)	72 (8.2)	895 (10.4)	1,115 (11.3)	19,703 (11.1)
	2008–2009	23,269 (10.9)	1,829 (11.3)	98 (11.2)	951 (11.0)	1,063 (10.8)	19,328 (10.9)
	2010–2011	22,979 (10.8)	1,797 (11.1)	119 (13.6)	1,026 (11.9)	1,214 (12.3)	18,823 (10.6)
	2012–2013	23,207 (10.9)	1,863 (11.5)	97 (11.1)	1,152 (13.4)	1,220 (12.4)	18,875 (10.7)
	2014–2015	23,466 (11.0)	1,787 (11.0)	145 (16.5)	1,156 (13.4)	1,185 (12.0)	19,193 (10.8)
	2016–2017	23,666 (11.1)	1,832 (11.3)	115 (13.1)	1,313 (15.2)	1,256 (12.7)	19,150 (10.8)
Health status & demographics							
NCI comorbidity index	Mean (SD)	0.3 (0.4)	0.4 (0.6)	0.4 (0.5)	0.3 (0.4)	0.3 (0.5)	0.3 (0.4)
Frailty score	Mean (SD)	0.2 (0.1)	0.2 (0.1)	0.2 (0.1)	0.2 (0.1)	0.2 (0.1)	0.2 (0.1)
Number of subsequent invasive primaries diagnosed during treatment period	Mean (SD)	0.03 (0.2)	0.03 (0.2)	0.03 (0.2)	0.03 (0.2)	0.03 (0.2)	0.03 (0.2)
Marital status	Married or domestic partner	90,605 (42.6)	3,981 (24.6)	316 (36.0)	4,344 (50.4)	3,958 (40.1)	78,006 (44.1)
	Single (never married)	16,469 (7.7)	2,384 (14.7)	100 (11.4)	701 (8.1)	1,078 (10.9)	12,206 (6.9)
	Previously married	94,576 (44.5)	8,856 (54.7)	368 (42.0)	3,227 (37.5)	4,310 (43.7)	77,815 (44.0)
	Unknown/missing	10,905 (5.1)	966 (6.0)	93 (10.6)	344 (4.0)	517 (5.2)	8,985 (5.1)
Low-income subsidy	Received	29,636 (13.9)	5,954 (36.8)	300 (34.2)	2,679 (31.1)	3,926 (39.8)	16,777 (9.5)
Guideline-concordance measures							
Diagnostic workup (includes HR status determination, HER2 status determination, pathology review, breast biopsy, and diagnostic mammography; indication based on metastatic status)	Concordant	174,666 (82.2)	12,503 (77.2)	680 (77.5)	7,070 (82.1)	7,902 (80.1)	146,511 (82.8)
	Non-concordant	37,889 (17.8)	3,684 (22.8)	197 (22.5)	1,546 (17.9)	1,961 (19.9)	30,501 (17.2)
Locoregional treatment (includes cancer-directed surgery, axillary staging, and radiation; indication based on T, N, and surgery type)	Concordant	146,718 (69.0)	10,155 (62.7)	576 (65.7)	6,281 (72.9)	6,763 (68.6)	122,943 (69.5)
	Non-concordant	36,601 (17.2)	3,096 (19.1)	155 (17.7)	1,365 (15.8)	1,656 (16.8)	30,329 (17.1)
	Undetermined*^a^	29,236 (13.8)	2,936 (18.1)	146 (16.7)	970 (11.3)	1,444 (14.6)	23,740 (13.4)
Systemic therapy (includes chemotherapy, HER2-targeted therapy, and hormone therapy; indication based on HR and HER2 status, stage at diagnosis, and tumor size)	Concordant	43,925 (20.7)	3,271 (20.2)	176 (20.1)	2,287 (26.5)	2491 (25.3)	35,700 (20.2)
	Non-concordant	10,659 (5.0)	984 (6.1)	63 (7.2)	483 (5.6)	584 (5.9)	8,545 (4.8)
	Undetermined*^b^	157,971 (74.3)	11,932 (73.7)	638 (72.8)	5,846 (67.9)	6,788 (68.8)	132,767 (75.0)

N and column percentages provided for categorical covariates; mean and standard deviation (SD) reported for continuous covariates. Statistical significance was tested via Chi-square tests for categorical and ANOVA for continuous covariates; all significantly varied by race and ethnicity group (*p* < 0.0001) except for number of subsequent tumors diagnosed (*p* = 0.06)

^*^Patients missing data necessary to characterize guideline-concordance were considered “undetermined” for that outcome

^a^Locoregional guideline-concordance is undetermined (i.e., unknown) for patients not meeting any of the following: T1-3, M0, known nodal status (positive or negative), not diffuse tumor

^b^Systemic guideline-concordance is undetermined (i.e., unknown) for patients not meeting any of the following criteria: diagnosed 2010 or later (when HER2 status data was first reported), continuous Part D prescription drug coverage for at least 3 months following diagnosis, known HR and HER2 status, known nodal stage, known tumor size, not diffuse tumor

The interaction between race and ethnicity and guideline-concordance was statistically significant across all analyses of diagnostic workup and locoregional treatment, while the interaction with systemic therapy initiation was only significant in some models (Online Resource 3).

### Diagnostic workup

#### Overall

About eighty percent of the study cohort received guideline-recommended diagnostic workup (*n* = 174,666; 82.2%; data not shown). Guideline-concordant diagnostics was associated with reduced hazard of death from breast cancer at 2 and 5 years after diagnosis in all analyses (Tables 3 and 4). Adjusted for year, age, tumor characteristics, health status, and demographics, patients with non-concordant diagnostics had 51% higher hazard of cancer death at 2 years than those with concordant care (HR (95% CI); 1.51 (1.42–1.60)). After additional adjustment for receipt of guideline-recommended locoregional treatment and systemic therapy, non-concordant diagnostic workup was associated with 1.33-times (CI 1.25–1.41) greater HR of breast cancer death 2-year post-diagnosis. Similar patterns were observed in 5-year analyses.

**Table 3:** Two-year hazard ratios by guideline-concordance overall and stratified by race and ethnicity

2-Year HRs updated	Analysis	Crude	M1: Year, Age, & Stage	M2: HR & HER2 + M1	M3: Health status, demographics + M2	M4: GCC + M3
Diagnostics	**Overall** *n* = 212,555	**2.76** **(2.67–2.87)**	**1.78** **(1.71–1.85)**	**1.57** **(1.48–1.66)**	**1.51** **(1.42–1.60)**	**1.33** **(1.25–1.41)**
	**Black** *n* = 16,187	**2.16** **(1.95–2.40)**	**1.65** **(1.48–1.84)**	**1.55** **(1.32–1.81)**	**1.53** **(1.30–1.79)**	**1.32** **(1.13–1.55)**
	**AIAN** *n* = 877	**1.78** **(1.06–3.01)**	1.18 (0.67–2.07)	1.09 (0.42–2.83)	1.13 (0.43–2.94)	0.92 (0.34–2.52)
	**API** *n* = 8,616	**2.21** **(1.76–2.79)**	**1.40** **(1.09–1.79)**	**1.47** **(1.04–2.07)**	**1.50** **(1.06–2.12)**	1.29 (0.91–1.83)
	**HW** *n* = 9,863	**2.34** **(1.96–2.79)**	**1.71** **(1.42–2.06)**	**1.65** **(1.24–2.19)**	**1.57** **(1.18–2.10)**	**1.45** **(1.09–1.93)**
	**NHW** *n* = 177,012	**2.88** **(2.76–3.00)**	**1.80** **(1.73–1.88)**	**1.56** **(1.46–1.67)**	**1.51** **(1.41–1.61)**	**1.33** **(1.24–1.42)**
Locoregional treatment	**Overall** *n* = 183,319	**3.08** **(2.92–3.26)**	**2.42** **(2.28–2.56)**	**2.43** **(2.29–2.57)**	**2.17** **(2.05–2.30)**	**2.10** **(1.98–2.23)**
	**Black** *n* = 13,251	**2.92** **(2.49–3.41)**	**2.54** **(2.16–3.00)**	**2.62** **(2.23–3.08)**	**2.41** **(2.04–2.83)**	**2.32** **(1.97–2.73)**
	**AIAN** *n* = 731	**5.04** **(2.18–11.67)**	**6.07** **(2.35–15.68)**	**5.88** **(2.20–15.74)**	**5.07** **(1.72–14.96)**	**6.47** **(2.12–19.75)**
	**API** *n* = 7,646	**2.79** **(1.98–3.93)**	**1.95** **(1.36–2.80)**	**1.92** **(1.32–2.77)**	**1.84** **(1.26–2.67)**	**1.72** **(1.18–2.50)**
	**HW** *n* = 8,419	**2.98** **(2.27–3.92)**	**2.23** **(1.67–2.97)**	**2.34** **(1.76–3.12)**	**2.20** **(1.64–2.94)**	**2.11** **(1.58–2.82)**
	**NHW** *n* = 153,272	**3.07** **(2.89–3.27)**	**2.38** **(2.23–2.55)**	**2.39** **(2.24–2.55)**	**2.14** **(2.00–2.28)**	**2.07** **(1.93–2.21)**
Systemic therapy	**Overall** *n* = 54,584	**2.31** **(2.12–2.52)**	**2.31** **(2.10–2.54)**	**1.95** **(1.77–2.15)**	**1.79** **(1.62–1.97)**	**1.67** **(1.51–1.84)**
	**Black** *n* = 4,255	**1.98** **(1.58–2.48)**	**2.19** **(1.65–2.73)**	**1.83** **(1.42–2.35)**	**1.70** **(1.31–2.19)**	**1.58** **(1.22–2.04)**
	**AIAN** *n* = 239	1.74 (0.63–4.79)	1.60 (0.50–5.19)	1.36 (0.35–5.25)	1.02 (0.20–5.13)	0.21 (0.03–1.68)
	**API** *n* = 2,770	**4.40** **(2.83–6.84)**	**4.49** **(2.72–7.40)**	**4.16** **(2.52–6.85)**	**3.82** **(2.27–6.43)**	**3.56** **(2.10–6.03)**
	**HW** *n* = 3,075	**2.35** **(1.63–3.37)**	**2.40** **(1.58–3.65)**	**2.20** **(1.44–3.35)**	**2.09** **(1.35–3.21)**	**1.87** **(1.20–2.90)**
	**NHW** *n* = 44,245	**2.26** **(2.05–2.50)**	**2.22** **(1.99–2.49)**	**1.88** **(1.68–2.10)**	**1.73** **(1.55–1.94)**	**1.63** **(1.45–1.82)**

Hazard of breast cancer death 2-year post-diagnosis for patients with non-concordant care compared to those with concordant care. Hazard ratios and 95% Confidence Intervals estimated from multivariable Cox proportional hazards models. Bolded values reflect statistical significance at the 5% significance level

Model 1: year of diagnosis, age at diagnosis, and stage at diagnosis

Model 2: hormone receptor (HR) status and Human Epithelial Growth Factor Receptor 2 (HER2) status + Model 1

Model 3: comorbidity, frailty, subsequent tumors, low income, and marital status + Model 2

Model 4: Model 3 + all guideline-concordance measures

- Diagnostics adjusted for locoregional treatment and systemic therapy concordance

- Locoregional treatment adjusted for diagnostics and systemic therapy concordance

- Systemic therapy adjusted for diagnostics and locoregional treatment concordance

*AIAN* American Indian/Alaska Native, *API* Asian or Pacific Islander, *GCC* guideline-concordant care, *HER2* human epithelial growth factor receptor 2, *HR* hormone receptor, *HW* Hispanic White, *M1* Model 1, *M2* Model 2, *M3* Model 3, *M4* Model 4, *NHW* non-Hispanic White

**Table 4:** Five-year hazard ratios by guideline-concordance overall and stratified by race and ethnicity

Outcome	Analysis	Crude	M1: Year, Age, & Stage	M2: HR & HER2 + M1	M3: Health status, demographics + M2	M4: GCC + M3
Diagnostics	**Overall** *n* = 212,555	**2.30** **(2.23–2.36)**	**1.60** **(1.55–1.65)**	**1.46** **(1.4–1.53)**	**1.42** **(1.36–1.49)**	**1.29** **(1.23–1.35)**
	**Black** *n* = 16,187	**1.85** **(1.71–2.01)**	**1.48** **(1.36–1.62)**	**1.40** **(1.24–1.59)**	**1.40** **(1.24–1.58)**	**1.23** **(1.08–1.39)**
	**AIAN** *n* = 877	**1.69** **(1.14–2.52)**	1.11 (0.73–1.69)	0.87 (0.44–1.70)	0.89 (0.45–1.77)	0.70 (0.34–1.45)
	**API** *n* = 8,616	**2.08** **(1.75–2.46)**	**1.35** **(1.13–1.62)**	**1.43** **(1.12–1.82)**	**1.44** **(1.13–1.84)**	**1.30** **(1.02–1.67)**
	**HW** *n* = 9,863	**2.00** **(1.75–2.28)**	**1.58** **(1.38–1.82)**	**1.64** **(1.33–2.02)**	**1.59** **(1.29–1.95)**	**1.48** **(1.21–1.82)**
	**NHW** *n* = 177,012	**2.36** **(2.29–2.44)**	**1.61** **(1.56–1.67)**	**1.46** **(1.38–1.53)**	**1.42** **(1.35–1.49)**	**1.29** **(1.22–1.36)**
Locoregional treatment	**Overall** *n* = 183,319	**2.37** **(2.28–2.46)**	**2.01** **(1.93–2.09)**	**2.02** **(1.94–2.11)**	**1.88** **(1.80–1.95)**	**1.83** **(1.76–1.90)**
	**Black** *n* = 13,251	**2.28** **(2.04–2.55)**	**2.10** **(1.87–2.36)**	**2.16** **(1.92–2.43)**	**2.02** **(1.80–2.27)**	**1.97** **(1.75–2.22)**
	**AIAN** *n* = 731	**2.34** **(1.31–4.19)**	**2.94** **(1.53–5.64)**	**3.20** **(1.63–6.30)**	**2.92** **(1.45–5.91)**	**2.92** **(1.42–5.98)**
	**API** *n* = 7,646	**2.31** **(1.84–2.91)**	**1.88** **(1.88–2.39)**	**1.86** **(1.46–2.37)**	**1.83** **(1.43–2.34)**	**1.79** **(1.40–2.29)**
	**HW** *n* = 8,419	**2.41** **(2.01–2.89)**	**1.98** **(1.64–2.40)**	**2.07** **(1.71–2.51)**	**1.97** **(1.63–2.39)**	**1.92** **(1.58–2.32)**
	**NHW** *n* = 153,272	**2.35** **(2.25–2.45)**	**1.98** **(1.89–2.07)**	**1.99** **(1.90–2.08)**	**1.84** **(1.76–1.93)**	**1.80** **(1.72–1.88)**
Systemic therapy	**Overall** *n* = 54,584	**2.05** **(1.92–2.19)**	**1.98** **(1.84–2.13)**	**1.78** **(1.66–1.92)**	**1.66** **(1.54–1.79)**	**1.56** **(1.45–1.68)**
	**Black** *n* = 4,255	**1.75** **(1.45–2.10)**	**1.82** **(1.49–2.23)**	**1.64** **(1.34–2.01)**	**1.54** **(1.26–1.89)**	**1.40** **(1.14–1.72)**
	**AIAN** *n* = 239	2.24 (0.94–5.32)	2.08 (0.77–5.65)	1.98 (0.68–5.82)	2.08 (0.60–7.26)	1.73 (0.38–7.81)
	**API** *n* = 2,770	**2.88** **(2.04–4.07)**	**2.60** **(1.78–3.82)**	**2.47** **(1.69–3.62)**	**2.36** **(1.61–3.46)**	**2.22** **(1.51–3.28)**
	**HW** *n* = 3,075	**2.29** **(1.75–3.00)**	**2.08** **(1.54–2.82)**	**2.09** **(1.53–2.84)**	**1.97** **(1.44–2.69)**	**1.80** **(1.31–2.47)**
	**NHW** *n* = 44,245	**2.02** **(1.87–2.18)**	**1.94** **(1.78–2.11)**	**1.74** **(1.60–1.89)**	**1.63** **(1.50–1.79)**	**1.54** **(1.42–1.68)**

Hazard of breast cancer death 5-year post-diagnosis for patients with non-concordant care compared to those with concordant care. Hazard ratios and 95% Confidence Intervals estimated from multivariable Cox proportional hazards models. Bolded values reflect statistical significance at the 5% significance level

Model 1: year of diagnosis, age at diagnosis, and stage at diagnosis

Model 2: hormone receptor (HR) status and Human Epithelial Growth Factor Receptor 2 (HER2) status + Model 1

Model 3: comorbidity, frailty, subsequent tumors, low income, and marital status + Model 2

Model 4: Model 3 + all guideline-concordance measures

- Diagnostics adjusted for locoregional treatment and systemic therapy concordance

- Locoregional treatment adjusted for diagnostics and systemic therapy concordance

- Systemic therapy adjusted for diagnostics and locoregional treatment concordance

*AIAN* American Indian/Alaska Native, *API* Asian or Pacific Islander, *GCC* guideline-concordant care, *HER2* human epithelial growth factor receptor 2, *HR* hormone receptor, *HW* Hispanic White, *M1* Model 1, *M2* Model 2, *M3* Model 3, *M4* Model 4, *NHW* non-Hispanic White

#### Stratified

**Fig. 2:**
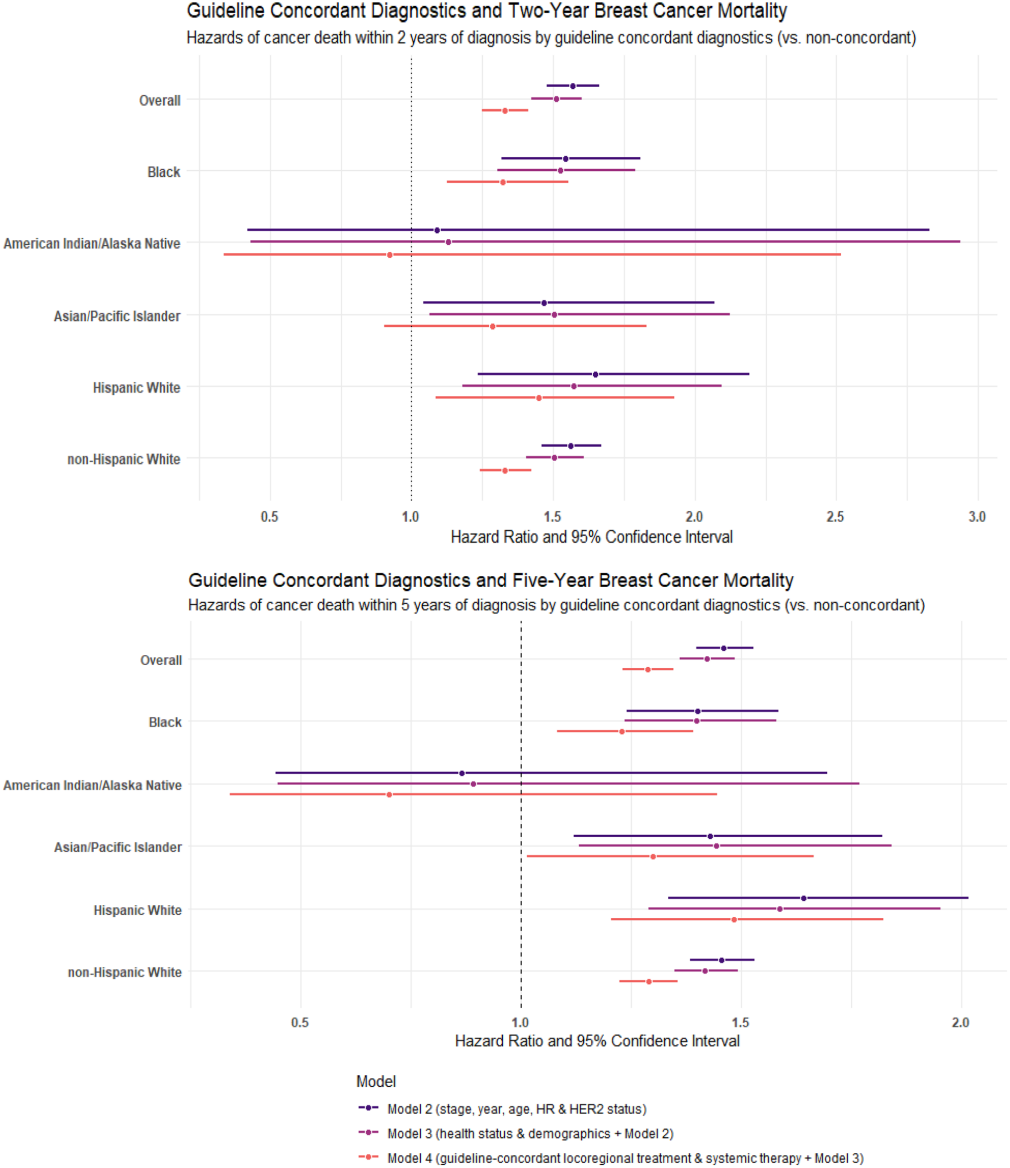

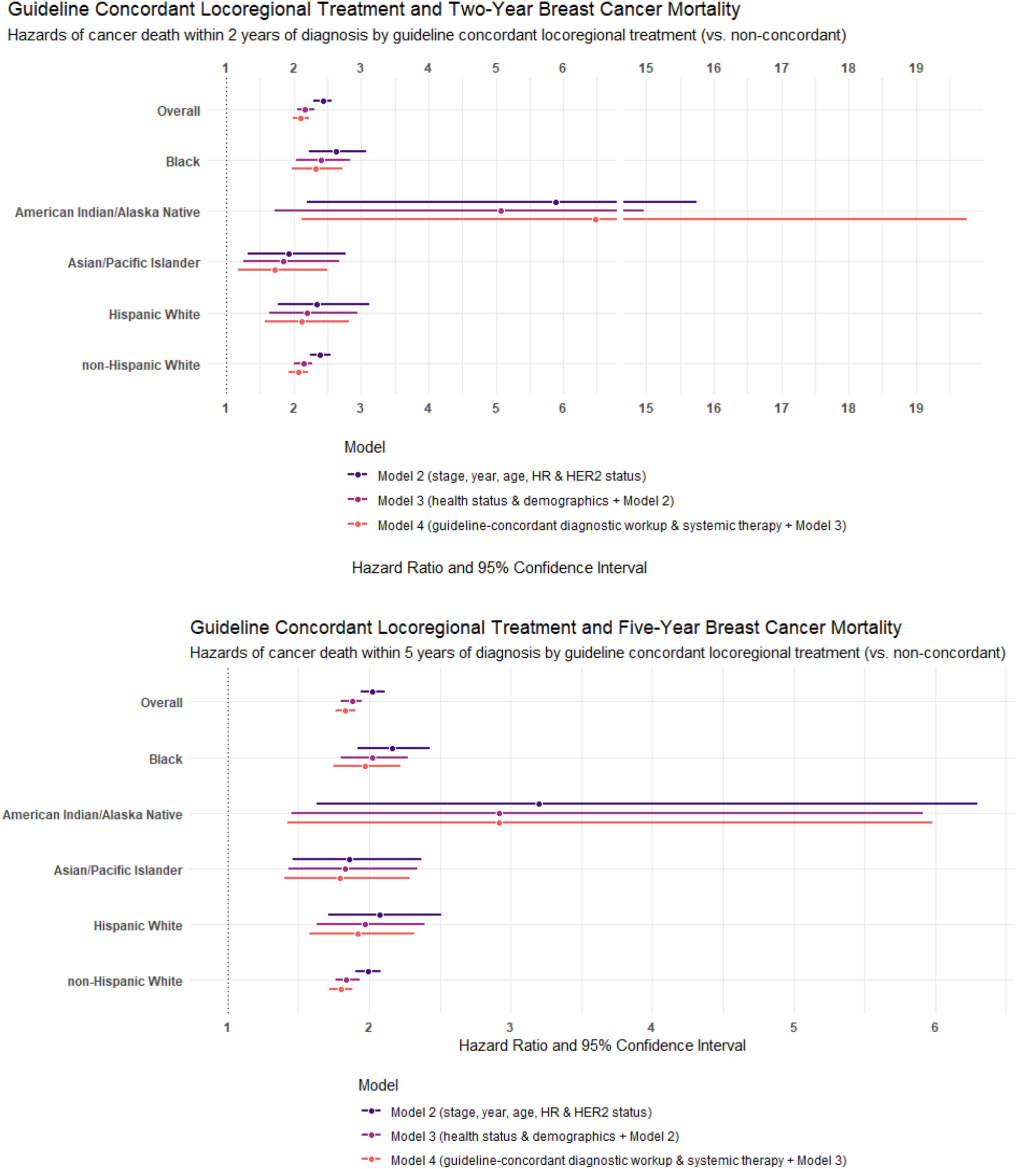

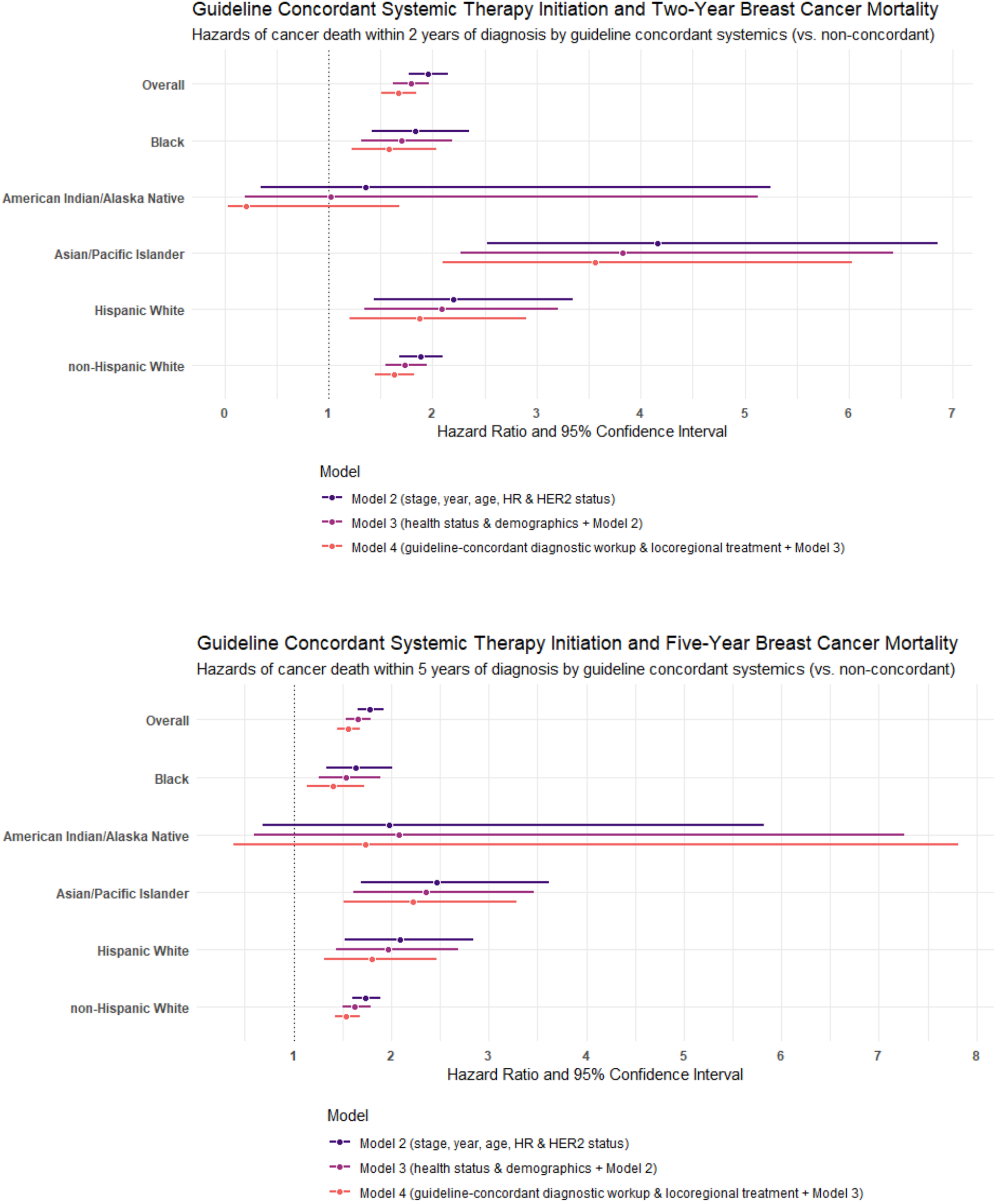
Hazard ratios for breast cancer mortality by guideline-concordant diagnostic workup, locoregional treatment, and systemic therapy initiation. Figures were created using R Statistical Software (v7.2.576; RRID:SCR_001905) via the ggplot2 R package (v2.0.6; RRID:SCR_014601)

Among AIAN patients, non-concordant diagnostics was associated with greater hazard of death at 2 and 5 years in unadjusted analyses, but there was no significant association after adjustment for covariates (Tables 3 and 4). AIAN women consistently had the lowest HRs for guideline-concordant diagnostics across analyses (Fig. 2).

Among Black women, non-concordant diagnostic workup was associated with higher hazard of breast cancer death in all analyses. Although Black patients had the lowest HRs after AIAN patients in crude analyses, their HRs were similar to other racial and ethnic groups in the fully adjusted model (HR_2-year, Model4_ 1.32 (1.13–1.55); HR_5-year, Model4_ 1.23 (1.08–1.39)).

Among API women, non-concordant diagnostics was associated with about 30% greater hazard of death at 2- and 5-year post-diagnosis after all adjustments (HR_2-year_ 1.29 (0.91–1.83); HR_5-year_ 1.30 (1.02–1.67)).

Hispanic White (HW) women had lower HRs than the overall cohort in the unadjusted model, but greater HRs in the fully adjusted model (HR_2-year, crude_ 2.34 (1.96–2.79); HR_2-year, Model_ 4 1.45 (1.09–1.93); HR_5-year, crude_ 2.00 (1.75–2.28); HR_2-year, Model 4_ 1.48 (1.21–1.82)).

Although non-Hispanic White (NHW) women had the largest HRs in the unadjusted models (HR_2-year_ 2.88 (2.76–3.00); HR_5-year_ 2.36 (2.29–2.44)), after all adjustments, non-concordant diagnostic workup was associated with an approximately 30% increased hazard of breast cancer death among NHW women at 2- and 5-year post-diagnosis (HR_2-year_ 1.33 (1.24–1.42); HR_5-year_ 1.29 (1.22–1.36)).

### Locoregional treatment

#### Overall

Eighty percent of the cohort eligible for locoregional treatment analyses received guideline-recommended care (*n* = 146,718; data not shown). Non-concordant locoregional treatment was associated with greater hazard of breast cancer death in all analyses (Tables 3 and 4). Adjusted for tumor characteristics, health status, and demographics, HRs of 2- and 5-year breast cancer death were 2.17 (2.05–2.30) and 1.88 (1.80–1.95), respectively, for patients with non-concordant versus concordant locoregional treatment. After all adjustments, patients who did not receive concordant locoregional treatment had 2.10- (1.98–2.23) and 1.83-times (1.76–1.90) greater hazard of breast cancer death at 2- and 5-year post-diagnosis.

#### Stratified

For all racial and ethnic groups other than AIAN, the magnitude of the hazards of breast cancer death by guideline-concordant locoregional treatment and patterns across adjusted models were similar to the overall cohort (Fig. 2).

AIAN patients had notably larger HRs, albeit with very wide confidence intervals, compared to the overall cohort and other racial and ethnic groups. AIAN patients receiving non-concordant locoregional treatment had approximately 6.5-times greater hazard of breast cancer death 2-year post-diagnosis compared to those with concordant care, with little variation across models (HR_Model4_ 6.47 (2.12–19.75); Table 3). At the 5-year endpoint, however, HRs for AIAN patients were of similar magnitude and followed a more similar pattern across models, although they still had the largest HR in the fully adjusted model (HR_Model4_ 2.92 (1.42–5.98); Table 4).

Black patients had larger HRs than most other racial and ethnic groups and the overall cohort at both 2- and 5-year endpoints in fully adjusted models (HR_2-year, Model4_ 2.32 (1.97–2.73); HR_5-year, Model4_ 1.97 (1.75–2.22)). API women had the smallest HRs for guideline-concordant locoregional treatment and breast cancer death across all models (HR_2-year, Model4_ 1.72 (1.18–2.50); HR_5-year, Model4_ 1.79 (1.40–2.29)). HW women receiving non-concordant locoregional treatment were approximately twice as likely to die from breast cancer as HW women receiving concordant locoregional treatment at 2- and 5-year post-diagnosis across adjusted models (HR_2-year, Model4_ 2.11 (1.58–2.82); HR_5-year, Model4_ 1.92 (1.58–2.32)).

Among NHW, unadjusted HRs for guideline-concordant locoregional treatment were greater than Black, API, and HW women at 2 years and greater than Black, AIAN, and API women at 5 years after diagnosis. However, after all adjustments, NHW had *smaller* HRs than the overall cohort and most racial and ethnic groups at both 2- and 5-year post-diagnosis.

### Systemic therapy initiation

#### Overall

Just over 80% of patients eligible for systemic therapy started guideline-recommended therapy (*n* = 43,925; 80.5%; data not shown). Two- and 5-year breast cancer death was greater for patients who did not initiate guideline-recommended systemics in all analyses (Tables 3 and 4). Adjusted for tumor characteristics, health status, and demographics, individuals initiating non-concordant systemic therapy had 1.79- (1.62–1.97) and 1.66-fold (1.54–1.79) greater hazard of breast cancer death at 2 and 5 years, respectively. With additional adjustment for guideline-recommended diagnostics and locoregional treatment, the HRs for breast cancer death associated with systemic therapy initiation were 1.67 (1.51–1.84) and 1.56 (1.45–1.68) at 2 and 5 years after diagnosis.

#### Stratified

Among AIAN patients, non-concordant systemic therapy initiation was not significantly associated with survival at 2- or 5-year post-diagnosis. The 2-year HR for systemics was close to the null when adjusted for tumor characteristics, health status, and demographics, but appeared protective with additional adjustment for guideline-recommended diagnostics and locoregional treatment (HR_Model3_ 1.02 (0.20–5.13); HR_Model4_ 0.21 (0.03–1.68)). At 5 years, the fully adjusted HR for breast cancer death among AIAN women was 1.73 (0.38–7.81).

Black patients had consistently smaller HRs for 2- and 5-year breast cancer death than the cohort overall across all analyses. In the fully adjusted models, non-concordant systemic therapy initiation was associated with HRs of 1.58 (1.22–2.04) at 2 years and 1.40 (1.14–1.72) at 5 years among Black women.

API patients had the largest HRs for the association between guideline-recommended systemic therapy initiation and breast cancer death in all analyses. Non-initiation of guideline-concordant systemics was associated with 3.56- (2.10–6.03) and 2.22-times (1.51–3.28) greater hazard of breast cancer death at 2 and 5 years after diagnosis in the fully adjusted model (Tables 3 and 4).

In most analyses of guideline-concordant systemics, HW patients had larger HRs than the overall cohort and most other racial and ethnic groups (HR_2-year, Model4_ 1.87 (1.20–2.90); HR_5-year, Model4_ 1.80 (1.31–2.47)). The magnitude and patterns across models for NHW patients were very similar to that of the overall cohort in both 2- and 5-year analyses.

### Sensitivity analysis

Results of our sensitivity analysis additionally adjusting for SEER registry in Models 3 and 4 are presented in Online Resource 4. There were no notable changes to the effect estimates in any analysis except those of AIAN women. For diagnostics and locoregional treatment, adjustment for SEER site resulted in larger changes to the magnitude of the HRs among AIAN women than any other group, although there were no changes in statistical significance. In the systemic therapy analyses, the effect estimates varied greatly in magnitude when adjusted for SEER site and became statistically significant despite very large confidence intervals at 5 years. Five-year HRs adjusted for health status, demographics, and tumor characteristics was 2.08 (0.60–7.26) in the primary analysis, increasing to 5.29 (1.19–23.61) with adjustment for SEER site. The fully adjusted 5-year HR among AIAN patients was 1.73 (0.38–7.81), which increased to 9.54 (1.15–79.27) with adjustment for SEER site. Of note, the fully adjusted 2-year systemics models including SEER site did not converge for AIAN women.

## Discussion

This novel investigation documented the impact of guideline-concordance along the breast cancer care continuum on survival in a diverse population-based cohort of older women in the U.S. Receipt of guideline-recommended diagnostic workup, locoregional treatment, and systemic therapy initiation were associated with reduced breast cancer death 2 and 5 years after diagnosis. Overall, we observed relatively consistent effects of guideline-recommended care from diagnosis through first-line treatment initiation on breast cancer mortality across racial and ethnic groups. While the magnitude of association varied, locoregional treatment was most strongly associated with survival for Black, AIAN, HW, and NHW patients, while systemic therapy initiation was more strongly associated with survival for API patients. Collectively, our findings demonstrate the universal importance of high-quality, guideline-adherent breast cancer care at every step of the breast cancer continuum, beginning at the time of diagnosis.

Our findings that locoregional treatment had the strongest effect on breast cancer death compared to diagnostic workup and systemic therapy initiation are consistent with other studies that have shown non-concordant treatment (surgery, radiotherapy, chemotherapy, or endocrine therapy) was associated with greater patient mortality risk compared to concordant treatment and relatively stronger effects for locoregional treatments compared to systemic therapies [65, 66]. To our knowledge, no studies have evaluated the role of receipt of the diagnostic workup in subsequent cancer survival. While the effect of non-concordant diagnostics on breast cancer death was modest (about 30% increased hazard of death), the association persisted despite adjustment for covariates including subsequent first-line treatment, suggesting quality during the initial phase of cancer care has a meaningful impact on patient outcomes.

Among AIAN patients, locoregional treatment had the greatest measured impact on 2- and 5-year survival; we observed no survival benefit related to guideline-recommended diagnostics and a smaller benefit from systemic therapy initiation among AIAN patients compared to other racial and ethnic groups. Logistics of receiving cancer care within or purchased by the Indian Health Service (IHS)—a federally funded healthcare system that provides care to eligible members of AIAN tribes—may explain these patterns [67]. Critically, IHS generally does not provide specialty medical care, including cancer treatment, and does not employ oncologists [26, 67, 68]. Rather, most AIAN patients must be referred for specialty care outside of the IHS. Thus, the lack of benefit of diagnostics among AIAN patients may be explained by complications of referral and care coordination across systems. Importantly, the magnitude of effects among AIAN patients was notably affected by adjustment for registry site. This could indicate the instability of the estimates due to small sample size and/or real area-based patterns of care contributing to survival disparities among Indigenous populations. In either case, this reinforces the importance of representation of Native peoples in cancer surveillance and research efforts.

Initiation of guideline-recommended systemic therapy had the largest impact on death among API patients, but a smaller effect among Black and AIAN women than the other racial and ethnic groups. These differences may be attributable to variability in adherence to or efficacy of systemic therapy in these groups, long-term adherence, completion of systemic therapy regimens, sub-optimal dosing, and whether it was provided in neoadjuvant and/or adjuvant settings [69–71]. Lower discontinuation and longer adherence to oral endocrine therapy and higher completion of HER2-targeted therapy have been documented among Asian patients [28, 72, 73]. Lower adherence to hormone therapy and HER2-targeted therapy for Black, compared to NHW, women has been previously demonstrated; however, two studies have reported no differences in hormone therapy adherence between Black and NHW women [28, 72–74]. These differences reflect social-structural barriers such as transportation, accessibility of pharmacies and healthcare facilities, and copays that hinder adherence to treatment plans among medically underserved populations, including Black and Indigenous populations [37, 75, 76].

Differences in the magnitude of the impact of guideline-concordant care on cancer death across racial and ethnic groups suggests provision of high-quality care alone is not wholly sufficient to eliminate survival disparities. Because our study is the first to examine the association between guideline-concordance and breast cancer death stratified by race and ethnicity, we cannot directly compare our findings to other literature. However, our findings for Black women are consistent with those from a recent study in Atlanta, Georgia which documented persistent disparities in breast cancer-specific survival after stratifying by guideline-concordant locoregional treatment and systemic therapy [77]. Understanding the underlying cause through a structural lens does not obviate intervention in cancer care or healthcare delivery settings, but demonstrates the importance of addressing the social risks and needs of patients created by structural barriers. Healthcare systems and institutions should consider interventions that directly address social and structural barriers faced by patients from medically underserved communities, such as food pantries in cancer clinics, coordinating transportation, and integrating services to facilitate patients’ navigation through the multi-disciplinary, complex course of cancer care.

Existing studies of race and ethnicity, guideline-concordant care, and breast cancer survival often treat race and ethnicity as a predictor or covariate in analyses, implicitly assuming differences are innate and immutable (biological or cultural). However, race and ethnicity are social constructs that reflect the pervasive influence of structural inequalities and social injustice that create unequal access to high-quality healthcare and are established root causes of poor health [76, 78]. With stratification by race and ethnicity, our estimates shed light on the relationship between quality of care and survival allowing variation between socially defined groups, rather than attempting to control these realities away. Additionally, this approach allows us to assess patterns among racial and ethnic groups in their own right, rather than solely in comparison to the most privileged (i.e., non-Hispanic Whites), in line with anti-colonial and antiracist research approaches [44–46, 79].

### Limitations

Race and ethnicity are not monoliths; aggregation of racial and ethnic groups obscures intra-group variation.

Our guideline-concordance care measures were limited to delivery of services and initiation of treatment based on the current guidelines at the time of beginning this analysis (NCCN guideline version 4.2021). Because the cohorts examined for each guideline-concordance measure were different and overlapping sample size was relatively small, we did not examine concordance across the continuum from diagnosis through systemic therapy initiation.

Follow-up time was measured from diagnosis which may yield immortal time bias since all patients were required to live at least three months past diagnosis. This design choice was made to ensure all patients lived long enough to receive some form of guideline-recommended care, but precludes generalization to patients who die quickly after diagnosis. Lead time and length biases may create artificially longer (i.e., more favorable) survival time among patients with screen-detected and/or slowly growing tumors [79, 81].

### Future directions

Further research should assess additional aspects of adherence to practice recommendations that were beyond the scope of this study, such as timing of adjuvant therapy, appropriateness of the regimen, and quality of treatment (e.g., negative margins status) [22, 23, 80]. Investigations of breast cancer survivorship disparities should also expand to include patient-centered outcomes such as patient satisfaction and quality of life, rather than just duration of survival time. For example, deployment of newer advances to clinical care such as OncotypeDX, which can indicate likelihood of response to chemotherapy, and help prevent unnecessary side effects and treatment burden where not essential for patient survival. In order to better understand how patterns of care quality influence survival across populations, more sophisticated modeling approaches should be deployed to disentangle the ways contextual factors modify these relationships. Further extension of this work to examine quality of care and survival across populations defined by factors such as Medicaid eligibility and/or receipt of low-income subsidies could provide critical insights to directly inform policy.

### Clinical and policy implications

These findings suggest the clinical practice guidelines are working as intended—achieving population-level survival benefits when care is delivered according to evidence-based recommendations. This indicates our critical challenge lies in increasing access to high-quality care for all patients—building capacity for high-quality, guideline-recommended care in community settings. The magnitude of the survival benefit of guideline-recommended locoregional treatment suggests this may be a pivotal point to focus supportive services and retention efforts.

These population-based findings may suggest a need to update care guidelines for resource limited settings—raising the bar for the minimum acceptable care in any clinical setting. For example, NCCN’s Framework which provides cancer care recommendations designed for resource limited settings based on available and affordable services in accordance with strength of the evidence.

## Conclusion

With documented disparities in evidence-based care beginning at the time of diagnosis, amelioration of survival disparities requires intentional effort to reduce barriers to high-quality care before, during, and after first-line treatment of breast cancer. This indicates the need for tailored and context-specific interventions that address barriers to accessing care pertinent to the individual and the phase of cancer care. Because racial and ethnic differences in quality of care are driven by social and structural factors, efforts to equitably improve access to high-quality care must be informed by, and responsive to, the fundamental root causes of disparities. To identify salient points of intervention, future research should explicitly examine the influence of the social and structural drivers of disparate quality of breast cancer care on survivorship.

## Supplementary Information

Below is the link to the electronic supplementary material.

Supplementary file1 (DOCX 136 KB)

Supplementary file2 (DOCX 44 KB)

Supplementary file3 (DOCX 16 KB)

Supplementary file4 (DOCX 24 KB)

## Data Availability

The SEER-Medicare datasets used to conduct this research are available to researchers upon approval of the research protocol by NCI and SEER. Instructions for obtaining these data are available at https://healthcaredelivery.cancer.gov/seermedicare/obtain/.
