# Supplementary material for: Guideline-concordance along the cancer care continuum and breast cancer mortality by race and ethnicity: a SEER-Medicare study
Source: Cancer Causes Control. 2026 Jan 21;37(2):33. doi: 10.1007/s10552-025-02099-9 (PMC12823728; doi:10.1007/s10552-025-02099-9)

**Online Resource 1: Kaplan-Meier Curves.** Figures created in SAS software version 9.4 (SAS Institute Inc., Cary, NC; RRID:SCR_008567).

**Figure A1. Diagnostic workup guideline-concordance: 2-Year Breast Cancer-Specific Mortality.** Blue denotes participants with non-concordant diagnostic workup. Red denotes participants with guideline-concordant diagnostic workup. The X axis is follow-up time from diagnosis in years, capped at 2 years from diagnosis. The Y axis is failure probability (1-survival probability) reflecting the cumulative probability of death due to breast cancer.


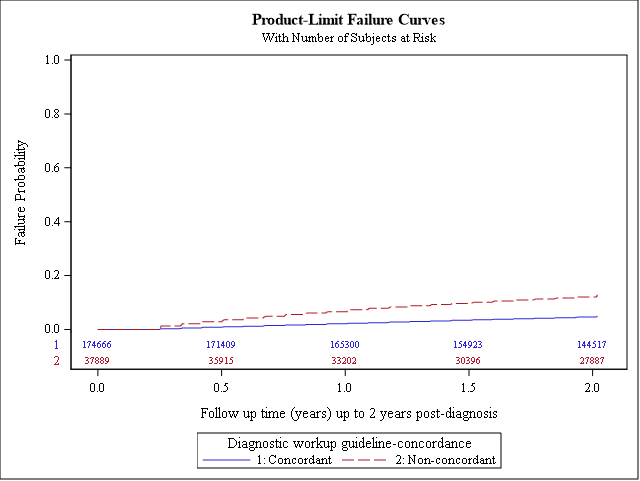


**Figure A2. Diagnostic workup guideline-concordance: 5-Year Breast Cancer-Specific Mortality.** Blue denotes participants with non-concordant diagnostic workup. Red denotes participants with guideline-concordant diagnostic workup. The X axis is follow-up time from diagnosis in years, capped at 5 years from diagnosis. The Y axis is failure probability (1-survival probability) reflecting the cumulative probability of death due to breast cancer.

**
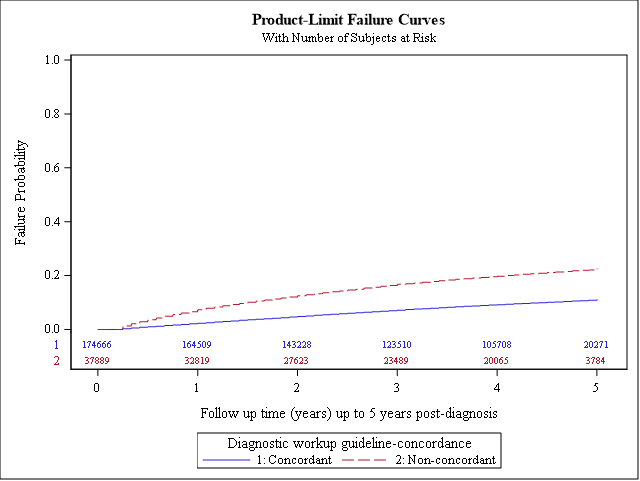
**

**Figure A3. Locoregional treatment guideline-concordance: 2-Year Breast Cancer-Specific Mortality** Blue denotes participants with non-concordant locoregional treatment. Red denotes participants with guideline-concordant locoregional treatment. Green denotes participants whose concordance with locoregional treatment guidelines was undetermined. The X axis is follow-up time from diagnosis in years, capped at 2 years from diagnosis. The Y axis is failure probability (1-survival probability) reflecting the cumulative probability of death due to breast cancer.

**
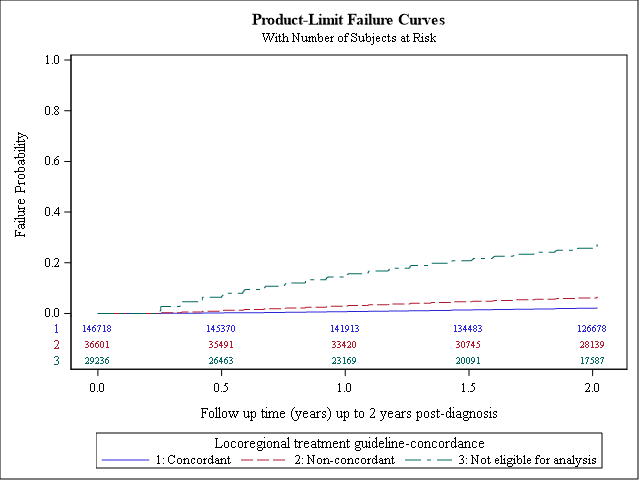
**

**Figure A4. Locoregional treatment guideline-concordance: 5-Year Breast Cancer-Specific Mortality.** Blue denotes participants with non-concordant locoregional treatment. Red denotes participants with guideline-concordant locoregional treatment. Green denotes participants whose concordance with locoregional treatment guidelines was undetermined. The X axis is follow-up time from diagnosis in years, capped at 5 years from diagnosis. The Y axis is failure probability (1-survival probability) reflecting the cumulative probability of death due to breast cancer.


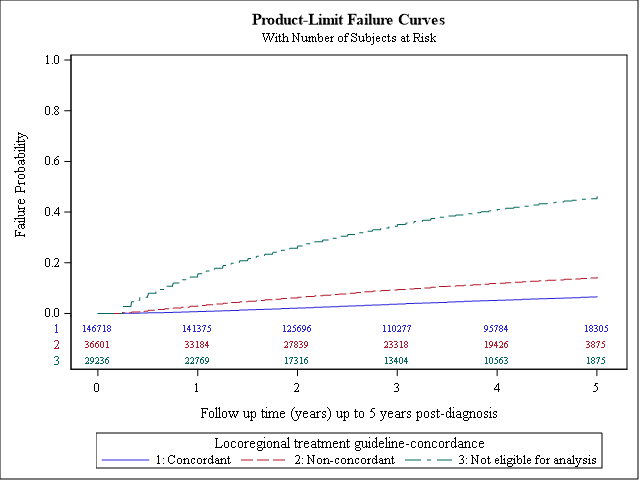


**Figure A5. Systemic therapy initiation guideline-concordance: 2-Year Breast Cancer-Specific Mortality.** Blue denotes participants with non-concordant systemic therapy initiation. Red denotes participants with guideline-concordant systemic therapy initiation. Green denotes participants whose concordance with systemic therapy guidelines was undetermined. The X axis is follow-up time from diagnosis in years, capped at 2 years from diagnosis. The Y axis is failure probability (1-survival probability) reflecting the cumulative probability of death due to breast cancer.


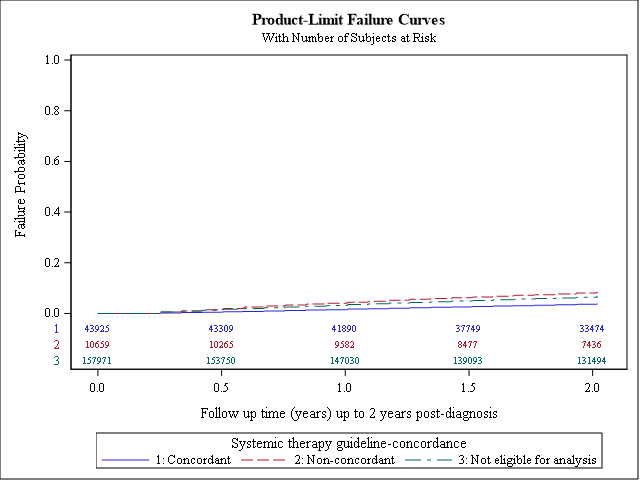


**Figure A6. Systemic therapy initiation guideline-concordance: 5-Year Breast Cancer-Specific Mortality.** Blue denotes participants with non-concordant systemic therapy initiation. Red denotes participants with guideline-concordant systemic therapy initiation. Green denotes participants whose concordance with systemic therapy guidelines was undetermined. The X axis is follow-up time from diagnosis in years, capped at 5 years from diagnosis. The Y axis is failure probability (1-survival probability) reflecting the cumulative probability of death due to breast cancer.


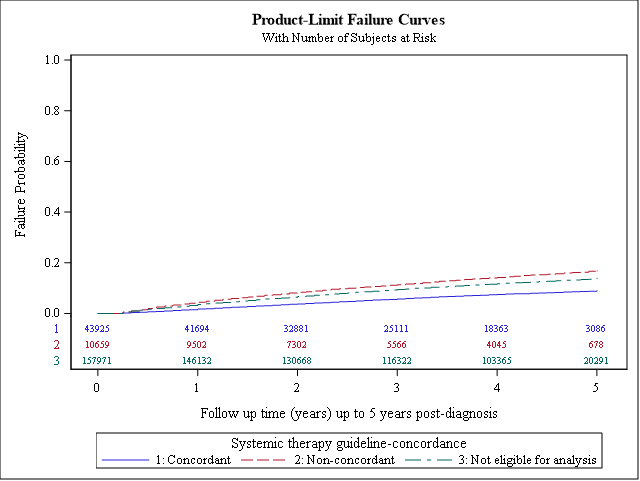

Supplement: Supplementary file 1 — Supplementary file1 (DOCX 136 KB) [file 10552_2025_2099_MOESM1_ESM.docx]
