# Supplementary material for: Guideline-concordance along the cancer care continuum and breast cancer mortality by race and ethnicity: a SEER-Medicare study
Source: Cancer Causes Control. 2026 Jan 21;37(2):33. doi: 10.1007/s10552-025-02099-9 (PMC12823728; doi:10.1007/s10552-025-02099-9)
Supplement: Supplementary file 2 — Supplementary file2 (DOCX 44 KB) [file 10552_2025_2099_MOESM2_ESM.docx]

**Online Resource 2: Cohort descriptives by outcome.**

**Table. Characteristics of Study Population by Guideline-Concordance Outcome**

N and column percentages provided for categorical covariates; mean and standard deviation (SD) reported for continuous covariates. Statistical significance was tested via Chi-square tests for categorical and ANOVA for continuous covariates; all significantly varied by each guideline concordance measure (p<0.05) except for the association between systemic therapy concordance and number of subsequent tumors diagnosed (p=0.063).

| **Covariate** | **Level** | **Diagnostics** | | | **Locoregional** | | | **Systemics** | | |
| --- | --- | --- | --- | --- | --- | --- | --- | --- | --- | --- |
|  |  | **Concordant** N=174,666 | | **Non-Concordant** N=37,889 | **Concordant** N=146,718 | **Non-Concordant** N=36,601 | **Not** **eligible** N=29,236 | **Concordant** N=43,925 | **Non-Concordant** N=10,659 | **Not eligible** N=157,951 |
| **Death endpoints** | | | | | | | | | | |
| Deaths within 2 years after diagnosis | Alive | 158,202  (90.6) | | 29,755  (78.5) | 138,377  (94.3) | 30,688  (83.8) | 18,892  (64.6) | 40,836  (93.0) | 8,870  (83.2) | 138,251  (87.5) |
|  | Cancer death | 7,919  (4.5) | | 4,429  (11.7) | 3,003  (2.1) | 2,163  (5.9) | 7,182  (24.6) | 1,527  (3.5) | 8,11  (7.6) | 10,010  (6.3) |
|  | Other (non-cancer) death | 8,545  (4.9) | | 3,705  (9.8) | 5,338  (3.6) | 3,750  (10.3) | 3,162  (10.8) | 1,562  (3.6) | 978  (9.2) | 9,710  (6.2) |
| Deaths within 5 years after diagnosis (or end of study data (12/31/2018), whichever comes first) | Alive | 136,406  (78.1) | | 23,066  (60.9) | 122,709  (83.6) | 24,255  (66.3) | 12,508  (42.8) | 37,562  (85.5) | 7,379  (69.2) | 114,531  (72.5) |
|  | Cancer death | 15,687  (9.0) | | 7,088  (18.7) | 7,781  (5.3) | 4,037  (11.0) | 10,957  (37.5) | 2,763  (6.3) | 1,281  (12.0) | 18,731  (11.9) |
|  | Other  (non-cancer) death | 22,573  (12.9) | | 7,735  (20.4) | 16,228  (11.1) | 8,309  (22.7) | 5,771  (19.7) | 3,600  (8.2) | 1,999  (18.8) | 24,709  (15.6) |
| Time to cancer death (years) | Mean (SD) | 3.8 (3.2) | | 3.0 (3.0) | 4.8 (3.4) | 3.5 (3.1) | 2.4 (2.4) | 2.4 (1.7) | 2.2 (1.7) | 3.8 (3.3) |
| Follow-up time (years) | Mean (SD) | 6.2 (4.3) | | 5.7 (4.7) | 6.6 (4.3) | 5.7 (4.5) | 4.0 (3.9) | 3.9 (2.2) | 3.6 (2.3) | 6.9 (4.6) |
| **Tumor characteristics** | | | | | | | | | | |
| Stage at diagnosis | I | 94,253  (54.0) | | 13,953  (36.8) | 86,294  (58.8) | 21,894  (59.8) | <20  (<0.1) | 24,968  (56.8) | <5,230  (<49.1) | 78,016  (49.4) |
|  | II | 52,158  (29.9) | | 10,048  (26.5) | 50,840  (34.7) | 11,356  (31.0) | <20  (<0.1) | 13,853  (31.5) | 3,665  (34.4) | 44,688  (28.3) |
|  | III | 14,109  (8.1) | | 4,833  (12.8) | 9,584  (6.5) | 3,351  (9.2) | 6,007  (20.6) | 3,122  (7.1) | 1,643  (15.4) | 14,177  (9.0) |
|  | IV | 7,167  (4.1) | | 1,860  (4.9) | 0 (0) | 0 (0) | 9,027  (30.9) | 1,899  (4.3) | 124  (1.2) | 7,004  (4.4) |
|  | Unknown/ missing | 6,979  (4.0) | | 7,195  (19.0) | 0 (0) | 0 (0) | 14,174  (48.5) | 83  (0.2) | <11  (<0.1) | 14,086  (8.9) |
| Hormone receptor (HR) status | Positive | 151,878  (87.0) | | 14,719  (38.9) | 119,890  (81.7) | 29,342  (80.2) | 17,365  (59.4) | 39,119  (89.1) | 8,885  (83.4) | 118,593  (75.1) |
|  | Negative | 22,788  (13.1) | | 2,821  (7.5) | 17,761  (12.1) | 4,149  (11.3) | 3,699  (12.7) | 4,806  (10.9) | 1,774  (16.6) | 19,029  (12.1) |
|  | Borderline or unknown | 0 (0) | | 20,349  (53.7) | 9,067  (6.2) | 3,110  (8.5) | 8,172  (28.0) | 0 (0) | 0 (0) | 20,349  (12.9) |
| Human Epidermal Growth Factor Receptor 2 (HER2) status | Positive | 8,856  (5.1) | | 679  (1.8) | 6,488  (4.4) | 1,490  (4.1) | 1,557  (5.3) | 3,951  (9.0) | 2,091  (19.6) | 3,493  (2.2) |
|  | Negative | 71,992  (41.2) | | 4,463  (11.8) | 56,212  (38.3) | 13,378  (36.6) | 6,865  (23.5) | 39,974  (91.0) | 8,568  (80.4) | 27,913  (17.7) |
|  | Borderline or unknown | 0 (0) | | 7,328  (19.3) | 3,148  (2.2) | 1,454  (4.0) | 2,726  (9.3) | 0 (0) | 0 (0) | 7,328  (4.6) |
|  | Data not available | 93,818  (53.7) | | 25,419  (67.1) | 80,870  (55.1) | 20,279  (55.4) | 18,088  (61.9) | 0 (0) | 0 (0) | 119,237  (75.5) |
| Triple negative subtype | Triple-negative | 7,109  (4.1) | | 592  (1.6) | 5,483  (3.7) | 1,207  (3.3) | 1,011  (3.46) | 3,488  (7.9) | 1,422  (13.3) | 2,791  (1.8) |
| Age at diagnosis | Mean (SD) | 75.7 (6.9) | | 77.8 (8.0) | 75.0 (6.3) | 78.7 (8.0) | 78.7 (8.2) | 74.6 (6.5) | 78.5 (7.7) | 76.4 (7.2) |
| Year of diagnosis | 2000-2001 | 16,837  (9.6) | | 7,447  (19.7) | 15,532  (10.6) | 4,583  (12.5) | 4,169  (14.3) | 0 (0) | 0 (0) | 24,284  (15.4) |
|  | 2002-2003 | 17,615  (10.1) | | 6,441  (17.0) | 15,211  (10.4) | 5,054  (13.8) | 3,791  (13.0) | 0 (0) | 0 (0) | 24,056  (15.2) |
|  | 2004-2005 | 19,559  (11.2) | | 4,516  (11.9) | 17,089  (11.7) | 3,418  (9.3) | 3,568  (12.2) | 0 (0) | 0 (0) | 24,075  (15.2) |
|  | 2006-2007 | 19,692  (11.3) | | 3,861  (10.2) | 16,760  (11.4) | 3,403  (9.3) | 3,390  (11.6) | 0 (0) | 0 (0) | 23,553  (14.9) |
|  | 2008-2009 | 20,115  (11.5) | | 3,154  (8.3) | 16,278  (11.1) | 3,821  (10.4) | 3,170  (10.8) | 0 (0) | 0 (0) | 23,269  (14.7) |
|  | 2010-2011 | 19,226  (11.0) | | 3,753  (9.9) | 16,121  (11.0) | 3,916  (10.7) | 2,942  (10.1) | 8,185  (18.6) | 2,571  (24.1) | 12,223  (7.7) |
|  | 2012-2013 | 20,042  (11.5) | | 3,165  (8.4) | 16,241  (11.1) | 4,132  (11.3) | 2,834  (9.7) | 10,412  (23.7) | 2,719  (25.5) | 10,076  (6.4) |
|  | 2014-2015 | 20,625  (11.8) | | 2,841  (7.5) | 16,640  (11.3) | 4,174  (11.4) | 2652  (9.1) | 12,225  (27.8) | 2,704  (25.4) | 8,537  (5.4) |
|  | 2016-2017 | 20,955  (12.0) | | 2,711  (7.2) | 16,846  (11.5) | 4,100  (11.2) | 2720  (9.3) | 13,103  (29.8) | 2,665  (25.0) | 7,898  (5.0) |
| **Health status & demographics** | | | | | | | | | | |
| NCI comorbidity index | Mean (SD) | 0.27 (0.4) | | 0.33 (0.5) | 0.25 (0.4) | 0.37 (0.5) | 0.35 (0.5) | 0.3 (0.4) | 0.4 (0.6) | 0.3 (0.4) |
| Frailty score | Mean (SD) | 0.15 (0.1) | | 0.16 (0.1) | 0.15 (0.1) | 0.17 (0.1) | 0.17 (0.1) | 0.16 (0.1) | 0.18 (0.1) | 0.15 (0.1) |
| Number of subsequent invasive primaries diagnosed during treatment period | Mean (SD) | 0.03 (0.2) | | 0.04 (0.2) | 0.03 (0.2) | 0.04 (0.2) | 0.04 (0.2) | 0.03 (0.2) | 0.04 (0.2) | 0.03 (0.2) |
| Marital status | Married or domestic partner | 77,527  (44.4) | | 13,078  (34.5) | 68,872  (46.9) | 13,153  (35.9) | 8,580  (29.4) | 20,197  (46.0) | 3,602  (33.8) | 66,806  (42.3) |
|  | Single (never married) | 13,137  (7.5) | | 3,332  (8.8) | 10,793  (7.4) | 2,942  (8.0) | 2,734  (9.4) | 4,138  (9.4) | 1,054  (9.9) | 11,277  (7.1) |
|  | Previously married | 76,041  (43.5) | | 18,535  (48.9) | 61,111  (41.7) | 18,588  (50.8) | 14,877  (50.9) | 17,490  (39.8) | 5,456  (51.2) | 71,630  (45.3) |
|  | Unknown/ missing | 7,961  (4.6) | | 2,944  (7.8) | 5,942  (4.1) | 1,918  (5.2) | 3,045  (10.4) | 2,100  (4.8) | 547  (5.1) | 8,258  (5.2) |
| Low-income subsidy | Received | 22,356  (12.8) | | 7,280  (19.2) | 17,705  (12.1) | 6,055  (16.5) | 5,876  (20.1) | 8,321  (18.9) | 2,743  (25.7) | 18,572  (11.8) |
| **Guideline-concordance measures** | | | | | | | | | | |
| **Diagnostic workup** (includes HR status determination, HER2 status determination, pathology review, breast biopsy, and diagnostic mammography; indication based on metastatic status) | Concordant | | - | - | 128,709  (87.7) | 28,356  (77.5) | 17,601  (60.2) | 42,270  (96.2) | 9,733  (91.3) | 122,663  (77.7) |
|  | Non-concordant | | - | - | 18,009  (12.3) | 8,245  (22.5) | 11,635  (39.8) | 1,655  (3.8) | 926  (8.7) | 35,308  (22.4) |
| **Locoregional treatment**  (includes cancer-directed surgery, axillary staging, and radiation; indication based on T, N, and surgery type) | Concordant | | 128,709  (73.7) | 18,009  (47.5) | - | - | - | 34,461  (78.5) | 6,922  (64.9) | 105,335  (66.7) |
|  | Non-concordant | | 28,356  (16.2) | 8,245  (21.8) | - | - | - | 6,509  (14.8) | 3,116  (29.2) | 26,976  (17.1) |
|  | Undetermined*^a^ | | 17,601  (10.1) | 11,635  (30.7) | - | - | - | 2,955  (6.7) | 621  (5.8) | 25,660  (16.2) |
| **Systemic therapy**  (includes chemotherapy, HER2-targeted therapy, and hormone therapy; indication based on HR and HER2 status, stage at diagnosis, and tumor size) | Concordant | | 42,270  (24.2) | 1,655  (4.4) | 34,461  (23.5) | 6,509  (17.8) | 2,955  (10.1) | - | - | - |
|  | Non-concordant | | 9,733  (5.6) | 926  (2.4) | 6,922  (4.7) | 3,116  (8.5) | 621  (2.1) | - | - | - |
|  | Undetermined*^b^ | | 122,663  (70.2) | 35,308  (93.2) | 105,335  (71.8) | 26,976  (73.7) | 25,660  (87.8) | - | - | - |

*Patients missing data necessary to characterize guideline-concordance were considered “undetermined” for that outcome.

^a^ Locoregional guideline-concordance is undetermined

(i.e., unknown) for patients not meeting any of the following: T1-3, M0, known nodal status

(positive or negative), not diffuse tumor.

^b^ Systemic guideline-concordance is undetermined

(i.e., unknown) for patients not meeting any of the following criteria: diagnosed 2010 or later

(when HER2 status data was first reported), continuous Part D prescription drug coverage for at least 3 months following diagnosis, known HR and HER2 status, known nodal stage, known tumor size, not diffuse tumor.

**Abbreviations**: ANOVA=analysis of variance; HER2=human epithelial growth factor receptor 2; HR=hormone receptor; M=metastatic staging; N=nodal involvement; NCI=National Cancer Institute; SD=Standard Deviation; T=tumor staging
