# Supplementary material for: Guideline-concordance along the cancer care continuum and breast cancer mortality by race and ethnicity: a SEER-Medicare study
Source: Cancer Causes Control. 2026 Jan 21;37(2):33. doi: 10.1007/s10552-025-02099-9 (PMC12823728; doi:10.1007/s10552-025-02099-9)
Supplement: Supplementary file 3 — Supplementary file3 (DOCX 16 KB) [file 10552_2025_2099_MOESM3_ESM.docx]

**Online Resource 3: Multiplicative interaction results.**

**Table. Significance of interaction terms from joint tests.** Bolded values are statistically significant at 0.05 alpha level. All models were the same as the primary analyses, except with the addition of a multiplicative interaction term for race and ethnicity with each guideline-concordance outcome, respectively.

| **2-year** | **Crude** | **1: Year, Age, & Stage** | **2: Stage, HR, HER2 + M1** | **3: Health status & demographics + M2** | **4: GCC + M3** |
| --- | --- | --- | --- | --- | --- |
| **Diagnostics** | **<.0001** | **<.0001** | **<.0001** | **<.0001** | **<.0001** |
| **Locoregional treatment** | **<.0001** | **<.0001** | **<.0001** | **<.0001** | **<.0001** |
| **Systemic therapy** | **<.0001** | **0.0064** | 0.2743 | 0.8059 | 0.8778 |
| **5-year** | **Crude** | **1: Year, Age, & Stage** | **2: Stage, HR, HER2 + M1** | **3: Health status & demographics + M2** | **4: GCC + M3** |
| **Diagnostics** | **<.0001** | **<.0001** | **<.0001** | **<.0001** | **<.0001** |
| **Locoregional treatment** | **<.0001** | **<.0001** | **<.0001** | **<.0001** | **<.0001** |
| **Systemic therapy** | **<.0001** | **0.0004** | **0.0296** | 0.6043 | 0.7658 |

Model 1: year of diagnosis, age at diagnosis, and stage at diagnosis

Model 2: hormone receptor (HR) status and Human Epithelial Growth Factor Receptor 2 (HER2) status + Model 1

Model 3: comorbidity, frailty, subsequent tumors, low income, and marital status + Model 2

Model 4: Model 3 + all guideline-concordance measures

- diagnostics adjusted for locoregional treatment and systemic therapy concordance

- locoregional treatment adjusted for diagnostics and systemic therapy concordance

- systemic therapy adjusted for diagnostics and locoregional treatment concordance
