# Supplementary material for: Guideline-concordance along the cancer care continuum and breast cancer mortality by race and ethnicity: a SEER-Medicare study
Source: Cancer Causes Control. 2026 Jan 21;37(2):33. doi: 10.1007/s10552-025-02099-9 (PMC12823728; doi:10.1007/s10552-025-02099-9)
Supplement: Supplementary file 4 — Supplementary file4 (DOCX 24 KB) [file 10552_2025_2099_MOESM4_ESM.docx]

**Online Resource 4: Sensitivity analysis adjusted for rurality and SEER registry.**

**Table 1. Two-Year Hazard Ratios by Guideline-Concordance Model Building with Additional Adjustment for SEER registry.** Hazard of breast cancer death 2-years post-diagnosis for patients with non-concordant care compared to those with concordant care. Hazard ratios and 95% Confidence Intervals estimated from multivariable Cox proportional hazards models. Bolded values reflect statistical significance at the 5% significance level.

| **2-Year HRs** | Analysis | **M3: Health status, demographics+M2 + SEER SITE** | **M4: GCC+M3 +SEER SITE** |
| --- | --- | --- | --- |
| **Diagnostics** | **Overall**  N=212,555 | **1.51**  **(1.42-1.60)** | **1.33**  **(1.25-1.41)** |
|  | **Black**  N=16,187 | **1.54**  **(1.31-1.80)** | **1.34**  **(1.14-1.58)** |
|  | **AIAN**  N=877 | 1.40  (0.52-3.76) | 1.19  (0.42-3.37) |
|  | **API**  N=8,616 | **1.60**  **(1.13-2.26)** | 1.38  (0.97-1.96) |
|  | **HW**  N=9,863 | **1.55**  **(1.16-2.07)** | **1.43**  **(1.07-1.91)** |
|  | **NHW**  N=177,012 | **1.50**  **(1.40-1.61)** | **1.33**  **(1.24-1.42)** |
| **Locoregional treatment** | **Overall**  N=183,319 | **2.19**  **(2.06-2.32)** | **2.11**  **(1.99-2.24)** |
|  | **Black**  N=13,251 | **2.45**  **(2.08-2.89)** | **2.37**  **(2.01-2.80)** |
|  | **AIAN**  N=731 | **5.31**  **(1.71-16.50)** | **6.24**  **(1.92-20.32)** |
|  | **API**  N=7,646 | **1.92**  **(1.32-2.80)** | **1.79**  **(1.23-2.60)** |
|  | **HW**  N=8,419 | **2.24**  **(1.67-2.99)** | **2.14**  **(1.60-2.87)** |
|  | **NHW**  N=153,272 | **2.15**  **(2.01-2.30)** | **2.08**  **(1.95-2.23)** |
| **Systemic therapy** | **Overall**  N=54,584 | **1.77**  **(1.61-1.96)** | **1.65**  **(1.50-1.82)** |
|  | **Black**  N=4,255 | **1.70**  **(1.32-2.20)** | **1.59**  **(1.22-2.07)** |
|  | **AIAN**  N=239 | 1.62  (0.21-12.72) | *Could not be estimated |
|  | **API**  N=2,770 | **3.74**  **(2.21-6.32)** | **3.39**  **(1.98-5.80)** |
|  | **HW**  N=3,075 | **2.08**  **(1.35-3.22)** | **1.85**  **(1.19-2.89)** |
|  | **NHW**  N=44,245 | **1.72**  **(1.54-1.93)** | **1.61**  **(1.44-1.80)** |

Model 3: SEER registry site, comorbidity, frailty, subsequent tumors, low income, and marital status + Model 2

Model 4: Model 3 + all guideline-concordance measures

- diagnostics adjusted for locoregional treatment and systemic therapy concordance

- locoregional treatment adjusted for diagnostics and systemic therapy concordance

- systemic therapy adjusted for diagnostics and locoregional treatment concordance

**Abbreviations:** AIAN=American Indian / Alaska Native; API=Asian or Pacific Islander; GCC=guideline-concordant care; HER2=human epithelial growth factor receptor 2; HR=hormone receptor; HW=Hispanic White; M1=Model 1; M2=Model 2; M3=Model 3; M4=Model 4; NHW=non-Hispanic White

**Table 2. Five-Year Hazard Ratios by Guideline-Concordance Model Building with Additional Adjustment for SEER registry.** Hazard of breast cancer death 2-years post-diagnosis for patients with non-concordant care compared to those with concordant care. Hazard ratios and 95% Confidence Intervals estimated from multivariable Cox proportional hazards models. Bolded values reflect statistical significance at the 5% significance level.

| **5-year HRs** | Analysis | **M3: Health status, demographics+M2 + SEER SITE** | **M4: GCC+M3 +SEER SITE** |
| --- | --- | --- | --- |
| **Diagnostics** | **Overall**  N=212,555 | **1.42**  **(1.36-1.49)** | **1.29**  **(1.23-1.35)** |
|  | **Black**  N=16,187 | **1.40**  **(1.24-1.59)** | **1.24**  **(1.09-1.40)** |
|  | **AIAN**  N=877 | 1.08  (0.54-2.16) | 0.87  (0.42-1.82) |
|  | **API**  N=8,616 | **1.49**  **(1.16-1.90)** | **1.35**  **(1.05-1.73)** |
|  | **HW**  N=9,863 | **1.56**  **(1.27-1.92)** | **1.46**  **(1.18-1.79)** |
|  | **NHW**  N=177,012 | **1.42**  **(1.35-1.49)** | **1.29**  **(1.22-1.36)** |
| **Locoregional treatment** | **Overall**  N=183,319 | **1.89**  **(1.81-1.96)** | **1.84**  **(1.77-1.91)** |
|  | **Black**  N=13,251 | **2.05**  **(1.82-2.30)** | **2.00**  **(1.78-2.25)** |
|  | **AIAN**  N=731 | **3.46**  **(1.64-7.30)** | **3.47**  **(1.62-7.41)** |
|  | **API**  N=7,646 | **1.86**  **(1.46-2.38)** | **1.83**  **(1.43-2.33)** |
|  | **HW**  N=8,419 | **2.02**  **(1.66-2.44)** | **1.96**  **(1.61-2.37)** |
|  | **NHW**  N=153,272 | **1.85**  **(1.77-1.94)** | **1.81**  **(1.73-1.89)** |
| **Systemic therapy** | **Overall**  N=54,584 | **1.66**  **(1.54-1.78)** | **1.55**  **(1.44-1.67)** |
|  | **Black**  N=4,255 | **1.54**  **(1.25-1.89)** | **1.38**  **(1.12-1.69)** |
|  | **AIAN**  N=239 | **5.29**  **(1.19-23.61)** | **9.54**  **(1.15-79.27)** |
|  | **API**  N=2,770 | **2.28**  **(1.55-3.37)** | **2.13**  **(1.43-3.16)** |
|  | **HW**  N=3,075 | **2.03**  **(1.47-2.79)** | **1.85**  **(1.34-2.56)** |
|  | **NHW**  N=44,245 | **1.63**  **(1.50-1.78)** | **1.54**  **(1.41-1.67)** |

Model 3: SEER registry site, comorbidity, frailty, subsequent tumors, low income, and marital status + Model 2

Model 4: Model 3 + all guideline-concordance measures

- diagnostics adjusted for locoregional treatment and systemic therapy concordance

- locoregional treatment adjusted for diagnostics and systemic therapy concordance

- systemic therapy adjusted for diagnostics and locoregional treatment concordance

**Abbreviations:** AIAN=American Indian / Alaska Native; API=Asian or Pacific Islander; GCC=guideline-concordant care; HER2=human epithelial growth factor receptor 2; HR=hormone receptor; HW=Hispanic White; M1=Model 1; M2=Model 2; M3=Model 3; M4=Model 4; NHW=non-Hispanic White
